# Dietary Quality and Relationships with Metabolic Dysfunction-Associated Fatty Liver Disease (MAFLD) among United States Adults, Results from NHANES 2017–2018

**DOI:** 10.3390/nu14214505

**Published:** 2022-10-26

**Authors:** Ting Tian, Jingxian Zhang, Wei Xie, Yunlong Ni, Xinyu Fang, Mao Liu, Xianzhen Peng, Jie Wang, Yue Dai, Yonglin Zhou

**Affiliations:** 1 Institute of Food Safety and Assessment, Jiangsu Provincial Center for Disease Control and Prevention, Nanjing 210009, China; 2 Department of Environment and Health, Jiangsu Provincial Center for Disease Control and Prevention, Nanjing 210009, China; 3 Department of Public Health and Preventive Medicine, Kangda College, Nanjing Medical University, Lianyungang 222000, China; 4 Department of Fundamental and Community Nursing, School of Nursing, Nanjing Medical University, Nanjing 211166, China

**Keywords:** nutrition, dietary quality indexes, epidemiology, MAFLD, NHANES

## Abstract

Metabolic dysfunction-associated fatty liver disease (MAFLD) is a new definition for the evidence of hepatic steatosis and metabolic dysfunctions. The specific role of the dietary factors in the development and progress of the disease are not well illuminated. Thus, we conducted this study on the associations between dietary quality assessed by five dietary quality indexes (Dietary Inflammatory Index, DII; Mediterranean diet, MED; Dietary Approach to Stop Hypertension, DASH; Alternate Healthy Eating Index diet, AHEI; Healthy Eating Indices, HEI) and MAFLD phenotypes. This study was extracted from the latest NHANES 2017–2018 wave. Demographic information, health status, lifestyles, and dietary habits were reported in the questionnaire. Multivariate logistic regression and multivariate ordinal logistic regression methods were applied to explore the associations between dietary quality indexes and MAFLD or MAFLD with liver fibrosis. The weighted prevalence of Non-MAFLD, MAFLD without fibrosis, and MAFLD with fibrosis were 47.05%, 36.67%, and 16.28%, respectively, at the cutoff value of a median Controlled Attenuation Parameter (CAP) 248 dB/m and a median Liver Stiffness Measurement (LSM) 6.3 kPa. When the diagnostic cutoff values of CAP changed to 285 dB/m, the weighted prevalence of Non-MAFLD, MAFLD without liver fibrosis, and MAFLD with fibrosis turned to 64.62%, 22.08%, and 13.30%, respectively. All five dietary quality indexes, including DII, HEI-2015, AHEI, DASH, and MED, were all significantly associated with MAFLD phenotypes. DII was positively associated with MAFLD phenotypes, while other four dietary quality indexes, including HEI-2015, AHEI, DASH, and MED, were significantly associated with lower risk of MAFLD phenotypes. MAFLD is becoming a threatening public health concern among adult Americans and dietary quality is markedly associated with MAFLD phenotypes.

## 1. Introduction

Metabolic dysfunction-associated fatty liver disease (MAFLD) is a new definition replacing nonalcoholic fatty liver disease (NAFLD) that was proposed by a panel of international experts from 22 countries by using a positive diagnosis rather than a “none”-disease rubric. The diagnostic criteria of MAFLD are based on evidence of hepatic steatosis in addition to one of the following three criteria, namely overweight/obesity, presence of type 2 diabetes mellitus, or evidence of metabolic dysregulation [1]. MAFLD diagnosis does not require the exclusion of patients with alcohol intake or other chronic liver diseases [2]. The prevalence of MAFLD in the United States populations was as high as 39.1% in the results from the 2017–2018 National Health and Nutrition Examination Survey (NHANES) [3]. MAFLD has rapidly become one of the leading causes of hepatocellular carcinoma and cirrhosis in developed countries [4]. From 1999 to 2016, cardiovascular and renal risks and diseases have become highly prevalent in adults with MAFLD [5]. In the Third National Health and Nutrition Examination Survey, MAFLD was associated with an increased risk of all-cause mortality and a higher risk of cardiovascular mortality [6].

In recent years, more and more evidence has proved that lifestyle health factors including non-smoking, non-drinking, and healthy diets are related to extensive health outcomes [7,8]. Dietary factors influence the pathogenesis of liver disease and a high-quality diet can be applied to the potential treatment measures. As for liver steatosis, one main etiopathogenesis is that inflammation and oxidative stress play a central role, while the excessive intake of fat and carbohydrates leads to endoplasmic reticulum stress, oxidative stress, and the activation of inflammatory bodies in hepatocytes [9]. Besides, the protective effects of bioactive compounds in foods with antioxidant and anti-inflammatory capacity such as fibers, monounsaturated and omega-3 fatty acids, and phytosterols can reduce the risk of developing of liver steatosis [10].

Indices reflecting overall diet quality are used globally in research to predict the risk of various diseases and metabolic disorders such as metabolic syndrome (MetS) [11] and non-alcoholic fatty liver disease (NAFLD) [12]. These scores measure either the adherence to certain dietary patterns, such as the Mediterranean diet (MED) [13], Dietary Approach to Stop Hypertension (DASH) [14], or Alternate Healthy Eating Index diet (AHEI) [15]; to country-specific dietary guidelines such as Healthy Eating Indices (HEI) [15]; or to the inflammatory potential of diets such as Dietary Inflammatory Index (DII) [16].

Due to short time since the definition of MAFLD, the specific roles of the dietary factors in the development and progress of the disease are not well evaluated. Thus, this study aimed to ascertain the associations between dietary quality and MAFLD.

## 2. Methods

### 2.1. Study Design and Population

NHANES, a program of the National Center for Health Statistics (NCHS), is designed to assess the health and nutritional status of adults and children in the United States. The survey investigated a nationally representative sample of about 5000 persons each year using complex sampling methods on their demographic information, physical examinations, biochemical and nutritional indicators, and lifestyle questionnaires [17]. This study was extracted from the latest NHANES 2017–2018 wave, which assessed liver function using ultrasound and vibration-controlled transient elastography (VCTE) for the first time in the survey. A median Controlled Attenuation Parameter (CAP) defined the liver steatosis and a median Liver Stiffness Measurement (LSM) defined the liver fibrosis.

Of the total 9254 participants enrolled in this survey cycle, 5948 were available for liver ultrasound transient elastography examinations. The elastography measurements were obtained in the NHANES Mobile Examination Center (MEC), using the FibroScan model 502 V2 Touch equipped with a medium (M) or extra-large (XL) wand (probe).

**Figure 1:**
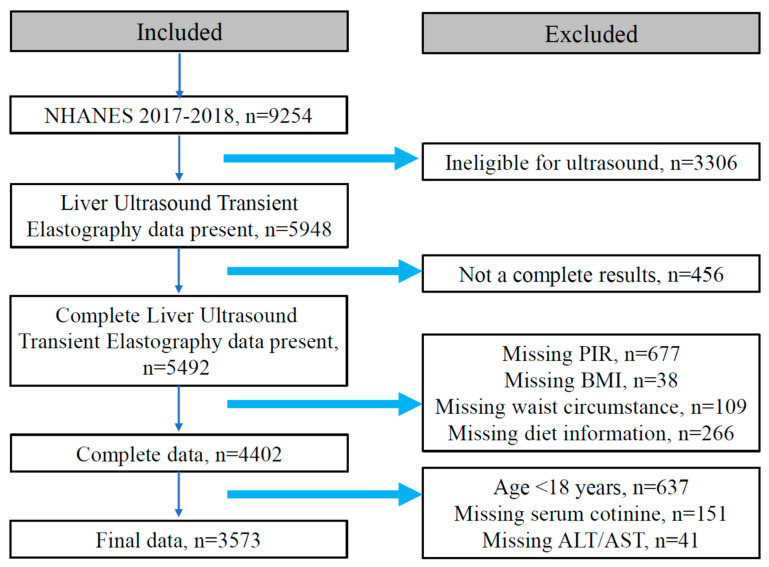
Flowchart of the selection in this study.

The exclusion criteria of this cross-sectional study were as follows: (1) not complete in the elastography exam (*n* = 456); (2) missing information of poverty income ratio (PIR) (*n* = 677), body mass index (BMI) (*n* = 38), waist circumstance (WC) (*n* = 109), diet information (*n* = 266); and (3) those who were aged less than 18 years old (*n* = 637), missing information of serum cotinine (*n* = 151), alanine aminotransferase (ALT), and aspartate aminotransferase (AST) (*n* = 41), displayed in Figure 1. Eventually, 3573 participants with complete demographic information, dietary information, anthropometric measurement results, and biomedical and nutritional examination indexes composed the study population. This survey was approved by the National Center for Health Statistics Research Ethics Review Board (Protocol number: 2018-01) and written informed consent was obtained from all participants.

### 2.2. Dietary Assessment and Covariates

Detailed dietary intake information is obtained from NHANES participants to estimate the energy, nutrients, and other food components from the foods consumed during the 24 h period prior to the interviews. All the participants are available for two 24 h dietary call interviews. The first interview is collected in face-to-face in the MEC and the second was conducted from 3 to 10 days later on the phone. Considering the reliability was higher in the in-person collection, we utilized the dietary information collected in the first interview to assess the diet quality and calculate the dietary quality indexes.

Demographic information, health status, and lifestyles were reported in the questionnaire including age, sex, race/ethnicity, education level, house income, physical activity, and smoking and drinking history. Race/ethnicity were categorized as Non-Hispanic White, Non-Hispanic Black, Mexican American, other Hispanic, and Other. Education levels were grouped into <high schools and ≥high schools. House income levels were defined by the poverty income ratio (PIR), which was low level (PIR < 1.30), middle level (1.30 ≤ PIR < 3.50), and high level (≥3.50) [18]. Physical activity (PA) was classified into low (<600 min/week), moderate (600 min/week–8000 min/week), and high levels (≥8000 min/week) using the metabolic equivalent of task (MET) (MET min/week) [19]. We classified smoking into three levels: low (serum cotinine < 0.015 ng/mL), moderate (0.015 ng/mL ≤ serum cotinine < 3 ng/mL), and high level (serum cotinine ≥ 3 ng/mL) [20]. In addition, alcohol consumption was classified into never drinkers, moderate drinkers (1–2 drinks/day for males, 1 drink/day for females), and heavy drinkers (≥2 drinks/day for males, ≥1 drink day for females) based on the definitions from US Department of Health and Human Services and US Department of Agriculture, 2020.

### 2.3. Diagnostic Criteria and Definitions

MAFLD: The proposed criteria for a positive diagnosis of MAFLD are based on histological (biopsy), imaging, or blood biomarker evidence of fat accumulation in the liver (hepatic steatosis) in addition to one of the following three criteria, namely overweight/obesity, presence of type 2 diabetes mellitus (T2DM), or evidence of metabolic dysregulation. The evidence of metabolic dysregulation is defined by the presence of at least two metabolic risk abnormalities, including (a) Waist circumference ≥ 102/88 cm in Caucasian men and women (or ≥90/80 cm in Asian men and women); (b) Blood pressure ≥ 130/85 mmHg or specific drug treatment: (c) Plasma triglycerides ≥ 150 mg/dL (≥1.70 mmol/L) or specific drug treatment; (d) Plasma HDL-cholesterol < 40 mg/dL (<1.0 mmol/L) for men and <50 mg/dL (<1.3 mmol/L) for women or specific drug treatment; (e) Prediabetes (i.e., fasting glucose levels 100 to 125 mg/dL [5.6 to 6.9 mmol/L], or 2 h post-load glucose levels 140 to 199 mg/dL [7.8 to 11.0 mmol] or HbA1c 5.7% to 6.4% [39 to 47 mmol/mol]); (f) Homeostasis model assessment of insulin resistance score ≥ 2.5; (g) Plasma high-sensitivity *C*-reactive protein level > 2 mg/L [1]. MAFLD phenotypes were defined as: non-MAFLD, MAFLD without clinical liver fibrosis, and MAFLD with clinical liver fibrosis.

Liver steatosis: CAP of 248 dB/m was used as the cutoff value to diagnose liver steatosis with sensitivity of 68.8%, specificity of 82.2%, and maximized Youden index [21]. Moreover, we also used another widely used cutoff value of 285 dB/m (sensitivity of 80% and specificity of 77%) in the diagnosis of hepatic steatosis for the consistency and reliability of our results [22]. Liver fibrosis: An optimal LSM cutoff of ≥6.3 kPa (with a sensitivity ≥ 90%) is indicative of clinical liver fibrosis [23].

Five dietary quality indexes: DII was designed and developed to compare the inflammatory potential of an individual’s diet based on 45 pro- and anti-inflammatory food parameters [16]. DII score represented diet inflammatory ability by combining the associations with the six inflammatory biomarkers: IL-1β, IL-4, IL-6, IL-10, TNF-α, and *C*-reactive protein. The higher the positive DII scores, the stronger the proinflammatory capacity, while the higher the negative DII value, the stronger the anti-inflammatory capacity. For the NHANES dietary questionaries, 28 of 45 food components were used to calculate the DII [24]. HEI-2015 is the updated version for assessing the fits between dietary intake and the new edition of the 2015–2020 Dietary Guidelines for Americans (DGA) (13 food components, 0–100 points) [25]. The HEI is based on density (e.g., quantities per 1000 kcal) rather than absolute quantities and relies on a common set of criteria applicable to individuals. The overall score of HEI represents overall diet quality and individual component scores that can be examined together to reveal patterns of quality across multiple dietary dimensions. The AHEI scores originated from 11 food components and points and are given on a scale from 0 to 110 [26]. All AHEI-2010 components were scored from 0 (worst) to 10 (best). This score, which emphasized high intakes of fruit, vegetables, and whole grains and low intakes of sodium and red and processed meats measures how diets most closely match the high-quality dietary guidelines. The DASH score is derived from eight components (total fat, saturated fat, protein, fiber, cholesterol, calcium, magnesium, and potassium) and is generated by the sum of all the nutrient target intakes (ranging from 8 points to 40 points) [27]. Mediterranean dietary patterns with a high intake of vegetables, fruits and nuts, and cereals, and a high intake of olive oil, a moderately high intake of fish, a low-to-moderate intake of dairy products and regular but moderate intake of red wines, have been proved to present beneficial health effects. The MED score ranging from 0 (minimal adherence to the traditional Mediterranean diet) to 9 (maximal adherence), is based on the nine indicated components [28].

### 2.4. Statistical Analysis

Continuous variables were displayed as a weighted mean (95% CI) and compared using the weighted linear regression analysis method. Categorical variables were described as a weighted percentage (95% CI) and compared by the Chi-square test. We used tertiles of five dietary quality indexes to compare the difference effects of high level, moderate level, and low level. Tertiles of DII were divided by the cutoff of 0.993 and 2.458. HEI-2015 was divided into tertiles by the value of 42.307 and 55.481. The cutoff values for AHEI tertiles were 42.607 and 52.921. The DASH tertiles were cut by the values of 24 and 27. MED was divided into three equal parts by the value of 6 and 7.

All statistical analyses were performed in R software (https://www.r-project.org/, accessed on 26 June 2022, 4.2.0 version, The R Foundation, Vienna, Austria). Appropriate sample weights were applied to represent the complex and multi-stage survey design of NHANES by the “Survey package”. Multivariate linear regression models were constructed to assess the relationship between various dietary quality indexes and CAP, LSM, ALT, and AST. Multivariate logistic regression and multivariate ordinal logistic regression methods were applied to explore the associations between dietary quality indexes and MAFLD or MAFLD with liver fibrosis. These models were adjusted for covariates, such as age, gender, race, education levels, PIR, physical activity levels, and smoking and drink conditions. It is considered that *p* < 0.05 is statistically significant and all statistical hypothesis tests are two-sided.

## 3. Results

### 3.1. Characteristics of Participants Based on MAFLD Phenotypes

A total of 3573 participants were finally included in this study. The overall characteristics of the study participants by category of MAFLD phenotypes were shown in Table 1. Significant differences existed in most demographic characteristics, biochemical indexes, anthropometric indexes, and dietary quality indexes among three MAFLD phenotypes. Those males and diabetes patients were more inclined to have more possibility of MAFLD or MAFLD with liver fibrosis. With the severity of MAFLD phenotypes, the age, CAP, LSM, BMI, WC, ALT, AST, Gamma-glutamyltransferase (GGT), Glycohemoglobin (GHB), Fasting Glucose (GLU), serum insulin, triglyceride (TG), Hypersensitive C Reactive Protein (HSCRP), and DII were increased (all *p* values < 0.05). The HEI-2015, AHEI and DASH were decreased with MAFLD phenotypes. In contrast, the education, PIR, smoking status, drinking status, and MED scores were not different among three MAFLD phenotypes (all *p* values > 0.05).

### 3.2. The Relationship between Various Dietary Quality Indexes and CAP, LSM, ALT, or AST

**Figure 2:**
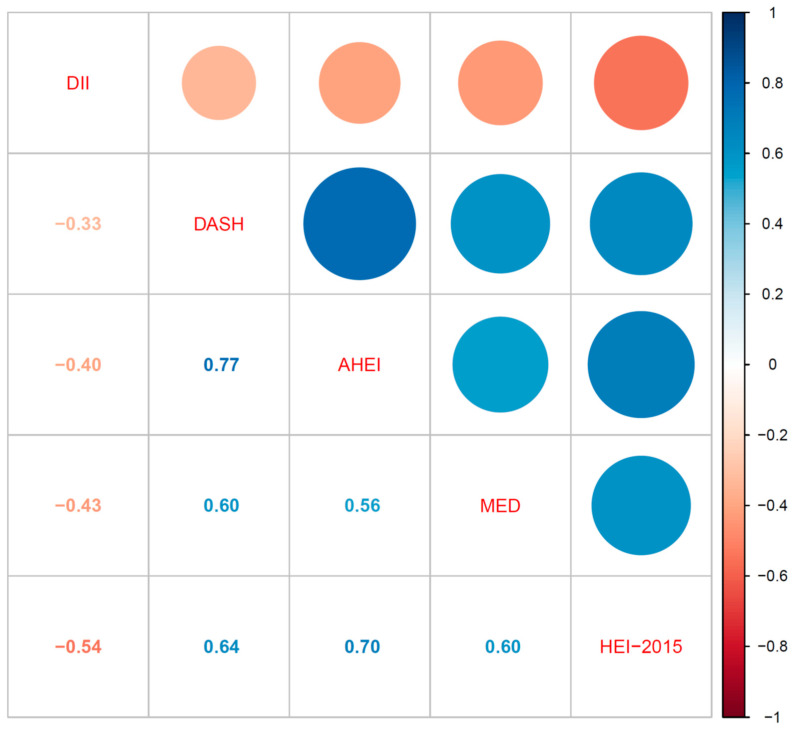
Pairwise correlations between five dietary quality indexes.

As shown in Figure 2, five dietary quality indexes turned out to be interrelated when assessed using correlation analysis (all *p* values <0.05). DII was negatively correlated with HEI-2015, AHEI, DASH, and MED. HEI-2015 was positively associated with AHEI, DASH, and MED. AHEI also had positive associations with DASH and MED. Additionally, there was a positive correlation between DASH and MED. Furthermore, we evaluated the relationship between five dietary quality indexes and liver function indexes using multivariate linear regression in Table 2. DII had a positive linear relationship with CAP, which the regression coefficient was 4.624 and the *p* value was 0.009. Moreover, HEI-2015, AHEI, and DASH were negatively related to CAP in a linear way (regression coefficients were -0.520, -0.605, and -2.112, respectively, and all *p* values <0.05). Except for HEI-2015, the other four dietary quality indexes were not related to LSM. ALT had no relationship with five dietary quality indexes. The other four dietary quality indexes were not correlated with AST, except for HEI-2015.

### 3.3. The Prevalence of MAFLD Phenotypes across Five Dietary Quality Indexes Tertiles

When the cutoff value for diagnosing liver steatosis was 248 dB/m, the weighted prevalence of Non-MAFLD, MAFLD without fibrosis, and MAFLD with fibrosis were 47.05%, 36.67%, and 16.28%, respectively, as displayed in Table 3. DII levels were not associated with the weighted prevalence of MAFLD phenotypes. HEI-2015, AHEI, DASH, and MED were inversely correlated with the weighted prevalence of MAFLD without or with liver fibrosis. Higher scores of HEI-2015, AHEI, DASH, and MED had a tendency to have less risk of MAFLD and liver fibrosis (all *p* for trend <0.05). Furthermore, in Supplementary Table S1, we used the 285 dB/m to diagnose liver steatosis, then the weighted prevalence of Non-MAFLD, MAFLD without liver fibrosis, and MAFLD with fibrosis turned to 64.62%, 22.08%, and 13.30%, respectively. Among higher diet qualities assessed by HEI-2015, AHEI, and MED scores, participants presented a lower risk of being prevalent of MAFLD without or with liver fibrosis (all *p* for trend <0.05).

### 3.4. Associations between Dietary Quality Indexes and MAFLD Phenotypes

We compared five dietary quality indexes with MAFLD risk using multivariate logistic regression and multivariate ordinal logistic regression and adjusted both with related covariates. From Table 4, in the multivariate logistic analysis, higher DII scores were associated with a higher risk of MAFLD when using DII as continuous scales (OR: 1.146, 95% CI: 1.041–1.260). However, this relationship did not exist in DII tertiles comparisons (both *p* > 0.05). When coming to HEI-2015, in the continuous scales, this index showed a protective effect against MAFLD (OR: 0.974, 95% CI: 0.968–0.990). T3 groups of HEI-2015 were less prone to having MAFLD compared to T1 groups (OR: 0.497, 95% CI: 0.335–0.738). Of note, AHEI, DASH, and MED were all negatively correlated with MAFLD risk on continuous scales (all adjusted ORs < 1 and all *p* < 0.05). Besides, compared with the T1 level of AHEI, DASH, and MED, the higher T2 and T3 levels were linked with reduced ORs of MAFLD (all adjusted ORs < 1 and all *p* < 0.05).

In addition, we subdivided three MAFLD phenotypes: non-MAFLD, MAFLD without clinical liver fibrosis, and MAFLD with clinical liver fibrosis. In the multivariate ordinal logistic regressions, all five dietary quality indexes, including DII, HEI-2015, AHEI, DASH, and MED, were all significantly associated with MAFLD phenotypes both in the continuous scales and tertile groups. Except for DII, which was positively associated with MAFLD phenotypes, the other four dietary quality indexes, including HEI-2015, AHEI, DASH, and MED, were significantly associated with a lower risk of MAFLD phenotypes. The adjusted ORs and 95% CI were 0.979 (0.970–0.988) for HEI-2015, 0.974 (0.966–0.982) for AHEI, 0.920 (0.898–0.943) for DASH, and 0.847 (0.756–0.949) for MED. Participants who were in T2 and T3 groups of HEI-2015, AHEI, DASH, and MED were less likely to be prevalent in MAFLD phenotypes (all adjusted ORs < 1 and all *p* < 0.05) compared to the T1 group. In addition, for the stability of the results, we also used another widely recognized diagnostic criteria (CAP: 285 dB/m) to diagnose liver steatosis. Additionally, we further assessed the relationships between five dietary indexes and MAFLD phenotypes by excluding heavy drinkers (*n* = 112). Similar results were revealed in the multivariate logistic analysis and multivariate ordinal analysis in Supplementary Table S2 and S3. Higher DII levels were still associated with a high risk of being MAFLD and MAFLD with liver fibrosis. In contrast, participants with high HEI-2015, AHEI, and DASH levels were less likely to be prevalent in MAFLD and MAFLD with liver fibrosis, whereas the MED levels did not reach statistical significance in the relationships to MAFLD and MAFLD phenotypes after the change of CAP diagnostic criteria.

## 4. Discussion

In 2020, a panel of international experts from 22 countries reached a consensus that it was suggested to use MAFLD to replace NAFLD because NAFLD did not reflect the current knowledge and metabolic dysfunction associated with liver diseases [1]. Diet quality had been verified to be associated with NAFLD in lots of research [12,29,30]. However, on the one hand, the mutual relationships of various dietary quality indexes were not fully understood and, on the other hand, associations between various dietary quality indexes and the newly proposed MAFLD were not comprehensively and systematically studied. Thus, this study utilized the representative sample of NHANES and found out that five dietary quality indexes turned out to be interrelated and that HEI-2015, AHEI, DASH, and MED were inversely correlated with the weighted prevalence of MAFLD without or with liver fibrosis. Furthermore, all five dietary quality indexes, including DII, HEI-2015, AHEI, DASH, and MED, were all significantly associated with MAFLD phenotypes. DII was positively associated with MAFLD phenotypes, while other four dietary quality indexes, including HEI-2015, AHEI, DASH, and MED, were significantly associated with a lower risk of MAFLD phenotypes. The main results of this study are summarized in Supplementary Figure S1 (summaries of the relationships between the five dietary indexes and MAFLD phenotypes).

The 2017 US Liver Disease Prevention and Control Guidelines put forward that obesity, type 2 diabetes, dyslipidemia, age, gender, and race were high-risk factors for fatty liver [31]. Similarly, we also found that males, diabetes patients, and elderly participants were more inclined to have serious MAFLD phenotypes. In addition, the levels of CAP, LSM, BMI, WC, ALT, AST, GGT, GLU, serum insulin, TG, and HSCRP were increased with the MAFLD phenotypes. These results exactly reflected the metabolic dysfunction (such as obesity, central obesity, dyslipidemia, abnormal liver function, and glucose metabolism disorder) of MAFLD. This study revealed that HEI-2015, AHEI, and DASH levels were decreased with MAFLD phenotypes. In a study aimed at NAFLD, the HEI-2015 levels were higher in the NAFLD patients than in the non-NAFLD patients [12]. Additionally, another study also showed that DASH and MED scores were inversely associated with NAFLD risk [32].

The diagnostic criteria for liver steatosis using CAP varied in different research. Thus, we selected two widely recognized cutoff values of CAP (248 dB/m and 285 dB/m). The weighted prevalence of MAFLD was 52.95% at the diagnostic criteria of 248 dB/m for CAP and 35.38% at 285 dB/m in this study, which were consistent with other recent results. Excellent concordance was noted between MAFLD and NAFLD diagnosis, a study came from the same database of 2017–2018 NHANES and revealed that 56.7% of the participants had NAFLD using CAP scores of ≥248 dB/m [33]. In a study of MAFLD in NHANES 2017–2018 ((*n* = 4328), 36.3% were prevalent with MAFLD using CAP scores of ≥285 dB/m) [34]. In a word, the US general adult population is facing a high risk and heavy disease burden of MAFLD.

Of note, with the high quality of diet assessed using HEI-2015, AHEI, DASH, and MED, the weighted prevalence of MAFLD without or with liver fibrosis decreased. In the current study, the inverse association between diet quality indexes and MAFLD and MAFLD with liver fibrosis risks were evident for HEI-2015, AHEI, DASH, and MED. In an updated meta-analysis, diets that score highly on the HEI, AHEI, and DASH were associated with a significant reduction in the risk of all-cause mortality, cardiovascular disease, cancer, type 2 diabetes, and neurodegenerative disease [15]. Longitudinal findings indicated that maintaining a high-quality diet during mid-to-late adulthood may prevent adverse metabolic consequences related to visceral adipose tissue (VAT) and nonalcoholic fatty liver (NAFL) [35]. However, the MED was not remarkable in the association with MAFLD and MAFLD phenotypes after we used the 285 dB/m of CAP as diagnostic criteria. The MED may be less sensitive to the variance in diet quality owing to its smaller range of total score (0–9 points) than the other indexes [28]. DII was found to be positively associated with MAFLD and MAFLD with liver fibrosis. The DII has been validated with various inflammatory markers and a higher and positive DII score indicates a more inflammatory diet [36]. It is now recognized that diet is an important modulator of chronic inflammation; the DII, characterizing the inflammatory potential of habitual diets, was associated with risks of a wide range of adverse health outcomes, such as cancer [37], cardiovascular diseases [38], and all-cause mortality [39].

The etiology of MAFLD is multifactorial and involves interactions between various factors, such as lifestyles, dietary factors, and individual inheritance and so on [1]. As for dietary influence, excessive caloric intake and nutritional patterns rich in saturated fat, carbohydrates, and sugar-sweetened beverages have all been implicated in the development of liver steatosis [40]. On one side, lipolysis of triglyceride increased free fatty acid. On the other side, the hepatocytes convert excess carbohydrates, especially fructose, to fatty acids. When the process of fatty acid disposal through beta-oxidation or generation of fatty acids is overloaded, the lipotoxic substances originate from fatty acids and lead to endoplasmic reticulum stress, oxidative stress, and the activation of inflammatory bodies [9]. Specific food or dietary compositions are also closely related to liver steatosis. For example, the over intake of saturated fatty acids will lead to hepatic gluconeogenesis, insulin resistance, and hepatic lipid accumulation [41]. Besides, high fructose intake will increase the accumulation of fats in the liver, the hepatic mRNA expression of fructokinase, and fat acids synthase. Additionally, fructose is involved in oxidative damage through the reduction in antioxidant defense and the improvement in the production of reactive oxygen species (ROS) resulting in necroinflammation [42]. Dietary fiber reduces the frequency of eating by intensifying satiety through the stimulation of the anorexigenic hormones and suppression of the orexigenic hormone ghrelin. This beneficial effect has been proved to be connected to weight reduction. Dietary supplementation with prebiotic can have a positive effect on NAFLD by modifying gut microbiota, reducing body fat, and bettering glucose regulation [43]. It is worth noticing that omega-3 polyunsaturated fatty acid signaling molecules can regulate liver lipid metabolism, activate the expression of enzymes involved in fat acid oxidation and suppress lipogenesis. Recently, omega-3 polyunsaturated fatty acids have been used as specific anti-steatosis drugs for NAFLD [44]. The fibrosis process is driven by signals from stressed or injured hepatocytes and activated macrophages (Kupffer cells in the liver), which lead to the activation of resident hepatic stellate cells into myofibroblasts to produce matrix proteins at a faster rate than degradation [45]. Diet may impact liver fat deposition by regulating overall adiposity. Several food components such as fruits and vegetables may affect liver fat by decreasing energy intake and increasing the production of beneficial short-chain fatty acids (SCFAs), which can suppress inflammation and thus reduce the risk of NAFLD [46]. Thus, high dietary quality, following specific dietary guidelines, such as Mediterranean Diet patterns with an appropriate proportion of energy (carbohydrates: 50–65% of total daily energy, fat: 30–35% of total energy with a high priority for monounsaturated fatty acids and omega-3 polyunsaturated fatty acids, protein: 15–20% of total daily energy), and a high intake of vegetables, fruits and nuts, cereals, olive oil, a moderately high intake of fish, a low-to-moderate intake of dairy products, and regular but moderate intake of red wines remains one of the mainstays in the management of patients with MAFLD.

Some merits of this study must be highlighted. We utilized the national representative large sample abiding by a rigorous and well-controlled protocol. Besides, five dietary quality indexes were comprehensively and systematically assessed in the relationship between diet and MAFLD phenotypes. Nonetheless, the limitations cannot be ignored in the current study. The cross-sectional design limited the ability to verify the inferences on causes and effects. In addition, dietary data were recorded using 24 h recall, thus, this recall data may not represent participants’ long-term dietary patterns. However, the questionnaire used to assess dietary intake in NHANES has been extensively validated against diet records and biomarkers [47] and we further adjusted the socioeconomic status, ethnic groups, and other characteristics. Overall, future randomized controlled trials and explorations of physiological mechanisms are required to confirm the effect of the change in each dietary score on the MAFLD phenotypes discovered in this study.

## 5. Conclusions

In conclusion, MAFLD is becoming a threatening public health problem among adult Americans. Five dietary quality indexes, including DII, HEI-2015, AHEI, DASH, and MED were all significantly associated with MAFLD phenotypes. Subjects who adhere to higher diet quality are less likely to have MAFLD and MAFLD with liver fibrosis. These results are of considerable public health importance for promoting health diet habitats in individuals and population groups, especially in countries with a high prevalence of MAFLD.

## Figures and Tables

**Table 1:** Basic information of the participants across MAFLD phenotypes.

Variables	Non-MAFLD Weighted Mean or Percentage	MAFLD without Clinical Fibrosis Weighted Mean or Percentage	MAFLD with Clinical Fibrosis Weighted Mean or Percentage	*p* Value
Gender				<0.001
Male	43.99	53.57	54.65	
Female	56.01	46.43	43.35	
Ethnicity				<0.001
Non-Hispanic White	66.75	64.24	65.44	
Non-Hispanic Black	11.20	8.16	9.50	
Other Hispanic	6.41	5.73	5.99	
Mexican American	5.90	11.34	10.28	
Other	9.75	10.54	8.78	
Education				0.562
<High school	9.53	10.03	11.09	
≥High school	90.47	89.97	88.91	
Diabetes				<0.001
No	96.01	85.42	61.95	
Yes	3.99	14.58	38.05	
PA level				<0.001
Low	22.75	33.74	35.92	
Moderate	56.36	47.96	45.85	
High	20.89	18.29	18.23	
PIR				0.680
Low	20.97	18.85	19.50	
Middle	33.46	36.65	37.93	
High	45.57	44.50	42.57	
Smoking Status				0.338
Low	35.94	39.85	41.20	
Moderate	37.85	37.655	36.44	
High	26.21	22.50	22.35	
Drink Status				0.093
Never	61.46	66.78	68.69	
Moderate	35.61	29.11	28.39	
Heavy	2.93	4.11	2.92	
Age (years)	42.70 (41.12–44.28)	50.13 (48.53–51.73)	52.01 (49.14–54.89)	<0.001
CAP (dB/m)	209.20 (207.53–210.87)	301.39 (298.77–304.01)	328.23 (321.77–224.69)	<0.001
LSM (kPa)	4.84 (4.65–5.03)	4.66 (4.58–4.73)	10.19 (9.16–11.22)	<0.001
BMI (kg/m^2^)	25.51 (24.98–26.03)	31.68 (30.75–32.02)	36.21 (35.01–37.41)	<0.001
WC (cm)	89.26 (88.03–90.49)	106.13 (104.67–107.60)	117.01 (114.80–119.23)	<0.001
Cotinine (ng/mL)	62.03 (47.38–76.69)	46.11 (39.75–52.46)	51.45 (31.31–71.59)	0.019
ALT (U/L)	19.70 (18.57–20.83)	24.34 (23.0–25.69)	32.38 (29.12–35.64)	<0.001
AST (U/L)	21.69 (20.54–2.84)	21.45 (20.67–22.22)	26.61 (24.58–28.64)	0.007
GGT (IU/L)	23.53 (22.08–24.99)	29.83 (27.75–31.90)	46.68 (40.06–53.30)	<0.001
GHB (%)	5.36 (5.32–5.39)	5.71 (5.63–5.79)	6.23 (6.13–6.33)	<0.001
GLU (mg/dL)	100.19 (99.21–101.18)	112.30 (108.83–115.78)	131.79 (123.15–140.43)	<0.001
Insulin (uU/mL)	4.04 (3.51–4.57)	7.29 (6.41–8.17)	11.69 (9.55–13.82)	<0.001
TG (mg/dL)	105.52 (100.79–11.026)	162.81 (154.69–170.93)	195.37 (179.45–211.29)	<0.001
HDL (mg/dL)	59.34 (57.78–60.89)	50.27 (49.14–51.41)	47.00 (45.30–48.71)	<0.001
HSCRP (mg/L)	2.54 (2.18–2.91)	4.22 (3.74–4.70)	5.60 (4.76–6.45)	<0.001
DII	1.36 (1.18–1.55)	1.50 (1.31–1.68)	1.64 (1.43–1.86)	0.032
HEI-2015	50.54 (48.40–52.69)	48.47 (47.10–49.85)	46.46 (44.84–48.08)	0.005
AHEI	49.00 (47.40–50.61)	47.01 (45.95–48.08)	45.24 (44.16–46.33)	<0.001
DASH	26.12 (25.56–26.68)	25.28 (24.90–25.65)	24.66 (24.18–25.14)	<0.001
MED	6.06 (5.91–6.21)	5.95 (5.84–6.06)	5.91 (5.79–6.07)	0.107

Abbreviations, CAP: a median Controlled Attenuation Parameter, LSM: a median Liver Stiffness Measurement, BMI: body mass index, WC: waist circumference, ALT: alanine aminotransferase, AST: aspartate aminotransferase, GGT: gamma-glutamyltransferase, GHB: glycohemoglobin, GLU: fasting blood glucose, TG: triglyceride, HDL: high-density lipoprotein, HSCRP: hypersensitive C reactive protein.

**Table 2:** Relationship between five dietary indexes and liver function indexes.

Variables	CAP		LSM		ALT		AST	
	Coefficients	*p* Value	Coefficients	*p* Value	Coefficients	*p* Value	Coefficients	*p* Value
DII	4.624	0.009	0.012	0.903	0.125	0.674	–0.128	0.471
HEI-2015	–0.520	0.013	–0.020	0.026	0.006	0.864	0.051	0.031
AHEI	–0.605	0.019	–0.018	0.061	–0.030	0.454	–0.002	0.875
DASH	–2.112	0.003	–0.034	0.320	–0.063	0.531	0.053	0.235
MED	–4.141	0.077	–0.062	0.485	0.389	0.450	0.337	0.381

Multivariate linear regression models adjusted for covariates, such as age, gender, race, education levels, PIR, physical activity levels, and smoking and drink conditions.

**Table 3:** The weighted prevalence of MAFLD phenotypes at the cutoff value 248 dB/m of CAP and 6.3 kPa of LSM.

Tertiles	Non-MAFLD (47.05%)	MAFLD without Clinical Fibrosis (36.67%)	MAFLD with Clinical Fibrosis (16.28%)	*p* Value	*p* for Trend
DII T1	49.84	35.85	14.30	0.494	0.016
T2	46.21	37.44	16.35		
T3	44.84	36.77	18.39		
HEI-2015 T1	42.13	37.72	20.14	0.002	0.001
T2	46.15	38.61	15.24		
T3	53.32	33.40	13.28		
AHEI T1	42.47	36.93	20.60	<0.001	<0.001
T2	46.46	37.93	15.62		
T3	52.51	35.15	12.34		
DASH T1	40.65	39.64	19.71	<0.001	<0.001
T2	46.30	37.02	16.69		
T3	52.56	34.11	13.34		
MED T1	43.44	38.95	17.61	0.329	0.008
T2	47.65	36.44	15.92		
T3	50.06	34.59	15.35		

**Table 4:** Relationship between five dietary indexes and MAFLD phenotypes at the cutoff value 248 dB/m of CAP and 6.3 kPa of LSM.

Dietary Quality Indexes	Multivariate Logistic Regression of MAFLD		Multivariate Ordinal Logistic Regression of MAFLD Phenotypes	
	OR (95%CI)	*p* Value	OR (95%CI)	*p* Value
DII				
Continuous scales	1.146 (1.041–1.260)	0.013	1.144 (1.069–1.225)	<0.001
T1 (Reference)	1.000		1.000	
T2	1.320 (0.982–1.774)	0.061	1.300 (1.058–1.593)	0.012
T3	1.568 (0.984–2.484)	0.056	1.561 (1.122–2.172)	0.008
HEI-2015				
Continuous scales	0.974 (0.968–0.990)	0.003	0.979 (0.970–0.988)	<0.001
T1 (Reference)	1.000		1.000	
T2	0.741 (0.531–1.034)	0.069	0.721 (0.576–0.902)	0.004
T3	0.497 (0.335–0.738)	0.006	0.510 (0.389–0.668)	<0.001
AHEI				
Continuous scales	0.974 (0.963–0.986)	0.002	0.974 (0.966–0.982)	<0.001
T1 (Reference)	1.000		1.000	
T2	0.722 (0.541–0.963)	0.034	0.698 (0.581–0.838)	<0.001
T3	0.535 (0.379–0.754)	0.005	0.519 (0.403–0.669)	<0.001
DASH				
Continuous scales	0.918 (0.892–0.945)	<0.001	0.920 (0.898–0.943)	<0.001
T1 (Reference)	1.000		1.000	
T2	0.743 (0.571–0.967)	0.034	0.765 (0.608–0.962)	0.022
T3	0.527 (0.397–0.699)	0.002	0.548 (0.437–0.688)	<0.001
MED				
Continuous scales	0.832 (0.719–0.962)	0.021	0.847 (0.756–0.949)	0.004
T1 (Reference)	1.000		1.000	
T2	0.737 (0.573–0.947)	0.026	0.759 (0.624–0.922)	0.005
T3	0.637 (0.433–0.939)	0.031	0.679 (0.519–0.887)	0.005

Multivariate logistic regression models and multivariate ordinal regression models (Non-MAFLD vs. MAFLD without fibrosis vs. MAFLD with fibrosis) were adjusted for covariates, such as age, gender, race, education levels, PIR, physical activity levels, and smoking and drink conditions.

## Data Availability

The datasets used and/or analyzed during the current study are available from the NHANES website (https://www.cdc.gov/nchs/nhanes/about_nhanes, accessed on 26 June 2022).

## Supplementary Materials

The following supporting information can be downloaded at: https://www.mdpi.com/article/10.3390/nu14214505/s1, Supplementary Table S1: The weighted prevalence of MAFLD phenotypes at the cutoff value 285 dB/m of CAP and 6.3 kPa of LSM. Supplementary Table S2. Relationship between five dietary indexes and MAFLD phenotypes at the cutoff value 285 dB/m of CAP and 6.3 kPa of LSM. Supplementary Table S3. Relationship between five dietary indexes and MAFLD phenotypes at the cutoff value 248 dB/m of CAP and 6.3 kPa of LSM without heavy drinkers. Supplementary Figure S1: Summaries of the relationships between five dietary indexes and MAFLD phenotypes.
