# Supplementary material for: Dietary Quality and Relationships with Metabolic Dysfunction-Associated Fatty Liver Disease (MAFLD) among United States Adults, Results from NHANES 2017–2018
Source: Nutrients. 2022 Oct 26;14(21):4505. doi: 10.3390/nu14214505 (PMC9659246; doi:10.3390/nu14214505)
Supplement: Supplementary file 1 [file nutrients-14-04505-s001.zip › nutrients-1990916-supplementary tables and figure legends.pdf]

**Supplementary Table S1**. The weighted prevalence of MAFLD phenotypes at the cutoff value 285 dB/m of CAP and 6.3 kPa of LSM**.**

| Tertiles | Non-MAFLD (64.62%) | MAFLD without fibrosis (22.08%) | MAFLD with fibrosis (13.30%) | *p* value | *p* for trend |
|---|---|---|---|---|---|
| DII T1 | 67.94 | 20.73 | 11.33 | 0.470 | 0.203 |
| T2 | 62.41 | 23.32 | 14.27 | | |
| T3 | 63.19 | 22.34 | 14.47 | | |
| HEI-2015 T1 | 61.79 | 22.03 | 16.18 | 0.069 | 0.027 |
| T2 | 63.56 | 23.94 | 12.50 | | |
| T3 | 68.81 | 20.13 | 11.06 | | |
| AHEI T1 | 61.33 | 22.21 | 16.46 | 0.019 | 0.022 |
| T2 | 64.35 | 22.58 | 13.07 | | |
| T3 | 68.38 | 21.47 | 10.15 | | |
| DASH T1 | 60.57 | 23.50 | 15.94 | 0.002 | 0.468 |
| T2 | 61.75 | 25.05 | 13.20 | | |
| T3 | 69.80 | 18.88 | 11.32 | | |
| MED T1 | 62.73 | 23.24 | 14.03 | 0.909 | 0.004 |
| T2 | 65.29 | 21.43 | 13.29 | | |
| T3 | 65.79 | 21.65 | 12.55 | | |

**Supplementary Table S2**. Relationship between five dietary indexes and MAFLD phenotypes at the cutoff value 285 dB/m of CAP and 6.3 kPa of LSM.

| **Dietary quality indexes** | | Multivariate logistic regression | | Multivariate ordinal logistic regression | |
|---|---|---|---|---|---|
| | | *OR (95%CI)* | *p* value | *OR (95%CI)* | *p* value |
| DII | | | | | |
| | Continuous scales | **1.137(1.012-1.276)** | **0.035** | **1.142(1.048-1.245)** | **0.002** |
| | T1(Reference) | 1.000 | | 1.000 | |
| | T2 | **1.446(1.052-1.989)** | **0.031** | **1.438(1.120-1.846)** | **0.004** |
| | T3 | 1.567(0.937-2.619) | 0.075 | **1.572(1.092-2.260)** | **0.015** |
| HEI-2015 | | | | | |
| | Continuous scales | **0.984(0.972-0.996)** | **0.018** | **0.982(0.973-0.992)** | **＜0.001** |
| | T1(Reference) | 1.000 | | 1.000 | |
| | T2 | 0.839(0.595-1.184) | 0.247 | **0.798(0.623-1.022)** | **0.074** |
| | T3 | **0.608(0.405-0.913)** | **0.025** | **0.596(0.448-0.794)** | **＜0.001** |
| AHEI | | | | | |
| | Continuous scales | **0.979(0.964-0.994)** | **0.013** | **0.978(0.967-0.989)** | **＜0.001** |
| | T1(Reference) | 1.000 | | 1.000 | |
| | T2 | 0.757(0.551-1.042) | 0.075 | **0.740(0.600-0.913)** | **0.005** |
| | T3 | **0.619(0.407-0.941)** | **0.032** | **0.594(0.437-0.808)** | **＜0.001** |
| DASH | | | | | |
| | Continuous scales | **0.933(0.892-0.975)** | **0.009** | **0.932(0.898-0.967)** | **＜0.001** |
| | T1(Reference) | 1.000 | | 1.000 | |

| | | | | |
|---|---|---|---|---|
| T2 | 0.900(0.671-1.208) | 0.400 | 0.873(0.697-1.094) | 0.238 |
| T3 | **0.576(0.383-0.867)** | **0.018** | **0.584(0.424-0.804)** | **0.001** |
| MED | | | | |
| Continuous scales | 0.873(0.727-1.048) | 0.119 | 0.873(0.755-1.010) | 0.067 |
| T1(Reference) | 1.000 | | 1.000 | |
| T2 | 0.809(0.560-1.170) | 0.200 | 0.806(0.591-1.100) | 0.174 |
| T3 | 0.765(0.464-1.259) | 0.226 | 0.769(0.536-1.103) | 0.153 |

Multivariate logistic regression models and multivariate ordinal regression models (Non-MAFLD *vs.* MAFLD without fibrosis *vs.* MAFLD with fibrosis) were adjusted for covariates, such as age, gender, race, education levels, PIR, physical activity levels, smoking and drink conditions.

**Supplementary Table S3**. Relationship between five dietary indexes and MAFLD phenotypes at the cutoff value 248 dB/m of CAP and 6.3 kPa of LSM without heavy drinkers

| **Dietary quality indexes** | Multivariate logistic regression | | Multivariate ordinal logistic regression | |
|---|---|---|---|---|
| | *OR (95%CI)* | *p* value | *OR (95%CI)* | *p* value |
| DII | | | | |
| Continuous scales | **1.152(1.049-1.265)** | **0.010** | **1.150(1.076-1.230)** | **＜0.001** |
| T1(Reference) | 1.000 | | 1.000 | |
| T2 | 1.328(0.978-1.802) | 0.063 | **1.306(1.055-1.617)** | **0.014** |
| T3 | **1.610(1.026-2.526)** | **0.042** | **1.607(1.162-2.223)** | **0.004** |
| HEI-2015 | | | | |
| Continuous scales | **0.978(0.968-0.989)** | **0.003** | **0.978(0.970-0.987)** | **＜0.001** |
| T1(Reference) | 1.000 | | 1.000 | |
| T2 | **0.710(0.508-0.993)** | **0.047** | **0.698(0.554-0.879)** | **0.002** |

| | | | | |
|---|---|---|---|---|
| T3 | **0.484(0.323-0.726)** | **0.006** | **0.497(0.377-0.655)** | **＜0.001** |
| AHEI | | | | |
| Continuous scales | **0.974(0.962-0.986)** | **0.002** | **0.973(0.965-0.981)** | **＜0.001** |
| T1(Reference) | 1.000 | | 1.000 | |
| T2 | **0.723(0.533-0.980)** | **0.041** | **0.693(0.575-0.835)** | **＜0.001** |
| T3 | **0.529(0.370-0.758)** | **0.006** | **0.510(0.393-0.661)** | **＜0.001** |
| DASH | | | | |
| Continuous scales | **0.919(0.894-0.945)** | **＜0.001** | **0.920(0.899-0.943)** | **＜0.001** |
| T1(Reference) | 1.000 | | 1.000 | |
| T2 | **0.741(0.570-0.962)** | **0.032** | **0.760(0.605-0.956)** | **0.019** |
| T3 | **0.532(0.406-0.698)** | **0.002** | **0.550(0.445-0.681)** | **＜0.001** |
| MED | | | | |
| Continuous scales | **0.824(0.712-0.953)** | **0.017** | **0.841(0.750-0.944)** | **0.003** |
| T1(Reference) | 1.000 | | 1.000 | |
| T2 | **0.758(0.591-0.974)** | **0.036** | **0.780(0.637-0.954)** | **0.016** |
| T3 | **0.618(0.417-0.916)** | **0.025** | **0.662(0.500-0.876)** | **0.004** |

Multivariate logistic regression models and multivariate ordinal regression models (Non-MAFLD *vs.* MAFLD without fibrosis *vs.* MAFLD with fibrosis) were adjusted for covariates, such as age, gender, race, education levels, PIR, physical activity levels, smoking and drink conditions. Heavy drinkers (n=112).

**Figure legends**

Supplementary Figure S1. Summaries of the relationships between five dietary indexes and MAFLD phenotypes. Model 1: Multivariate logistic regression analysis (MAFLD vs. Non-MAFLD), Model 2: Multivariate ordinal logistic regression analysis (MAFLD with fibrosis vs. MAFLD without fibrosis vs. non-MAFLD).
