# Supplementary figures and images for: Dietary Quality and Relationships with Metabolic Dysfunction-Associated Fatty Liver Disease (MAFLD) among United States Adults, Results from NHANES 2017–2018
Source: Nutrients. 2022 Oct 26;14(21):4505. doi: 10.3390/nu14214505 (PMC9659246; doi:10.3390/nu14214505)

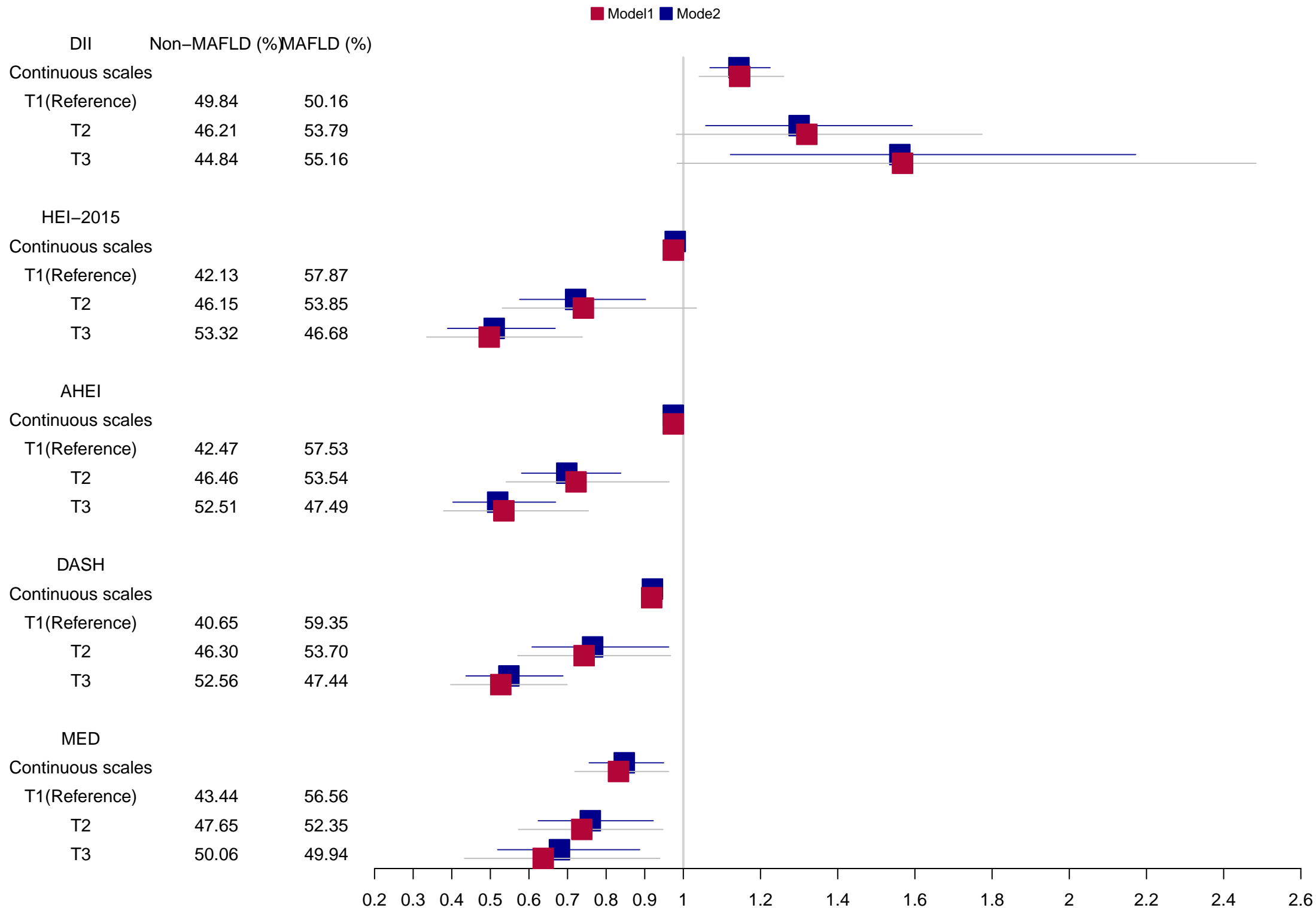

Supplement: Supplementary file 1 [file nutrients-14-04505-s001.zip › Supplementary Figure S1 Summaries of the relationships between five dietary indexes and MAFLD phenotypes.pdf]
